# Combined use of direct analysis in real-time/Orbitrap mass spectrometry and micro-Raman spectroscopy for the comprehensive characterization of real explosive samples

**DOI:** 10.1007/s00216-016-9691-9

**Published:** 2016-06-18

**Authors:** Maxime C. Bridoux, Adrián Schwarzenberg, Sébastien Schramm, Richard B. Cole

**Affiliations:** CEA, DAM, DIF, 91297 Arpajon, France; UPMC, IPCM/CSOB, UMR 8232, 4 Place Jussieu, 75252 Paris cedex 05, France

**Keywords:** Direct Analysis in Real Time, Orbitrap MS, Raman microscopy, Real weapons, Explosives, Polymers

## Abstract

Direct Analysis in Real Time (DART™) high-resolution Orbitrap™ mass spectrometry (HRMS) in combination with Raman microscopy was used for the detailed molecular level characterization of explosives including not only the charge but also the complex matrix of binders, plasticizers, polymers, and other possible organic additives. A total of 15 defused military weapons including grenades, mines, rockets, submunitions, and mortars were examined. Swabs and wipes were used to collect trace (residual) amounts of explosives and their organic constituents from the defused military weapons and micrometer-size explosive particles were transferred using a vacuum suction-impact collection device (vacuum impactor) from wipe and swap samples to an impaction plate made of carbon. The particles deposited on the carbon plate were then characterized using micro-Raman spectroscopy followed by DART-HRMS providing fingerprint signatures of orthogonal nature. The optical microscope of the micro-Raman spectrometer was first used to localize and characterize the explosive charge on the impaction plate which was then targeted for identification by DART-HRMS analysis in both the negative and positive modes. Raman spectra of the explosives TNT, RDX and PETN were acquired from micrometer size particles and characterized by the presence of their characteristic Raman bands obtained directly at the surface of the impaction plate nondestructively without further sample preparation. Negative mode DART-HRMS confirmed the types of charges contained in the weapons (mainly TNT, RDX, HMX, and PETN; either as individual components or as mixtures). These energetic compounds were mainly detected as deprotonated species [M–H]^−^, or as adduct [M + ^35^Cl]^−^, [M + ^37^Cl]^−^, or [M + NO_3_]^−^ anions. Chloride adducts were promoted in the heated DART reagent gas by adding chloroform vapors to the helium stream using an “in-house” delivery method. When the polarity was switched to positive mode, DART-HRMS revealed a very complex distribution of polymeric binders (mainly polyethylene glycols and polypropylene glycols), plasticizers (e.g., dioctyl sebacate, tributyl phosphate), as well as wax-like compounds whose structural features could not be precisely assigned. In positive mode, compounds were identified either as protonated molecules or ammonium adduct species. These results clearly demonstrate the complementarity of micro-Raman microscopy combined with DART-MS. The former technique provides structural information on the type of explosives present at the surface of the sample, whereas the latter provides not only a confirmation of the nature of the explosive charge but also useful additional information regarding the nature of the complex organic matrix of binders, plasticizers, polymers, oils, and potentially other organic additives and contaminants present in the sample. Combining these two techniques provides a powerful tool for the screening, comprehensive characterization, and differentiation of particulate explosive samples for forensic sciences and homeland security applications.

**Electronic supplementary material:**

The online version of this article (doi:10.1007/s00216-016-9691-9) contains supplementary material, which is available to authorized users.

**Graphical Abstract:**
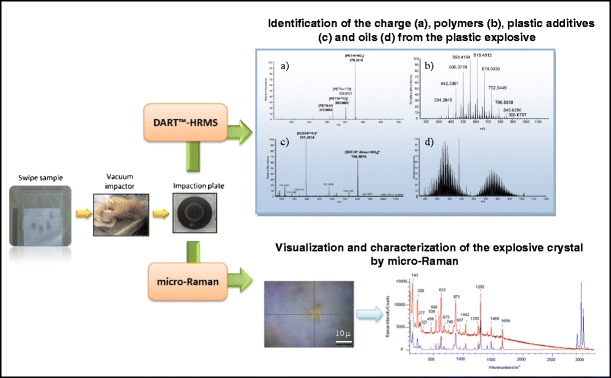
Comprehensive characterization of explosive particles collected from swipe samples by micro-Raman and DART™-HRMS

## Introduction

The ability to fully characterize at the molecular level a formulation of explosives from trace amounts of samples is vital for homeland security and forensic applications. Most explosives are composed of a charge which consists of a single or a mixture of energetic compounds such as trinitrotoluene (TNT), hexogen (RDX), nitropentaerythritol (PETN), plus a complex matrix of binders, plasticizers, polymers, oils, and potentially other organic additives and contaminants. The detailed characterization of the “matrix” surrounding the charge can give significant clues to identifying the geographic origin of the explosive, the manufacturing process, and perhaps even the lot number [1]. In order to confront terrorist activities and to address issues relevant to the forensic community there is an urgent need to develop techniques that allow the reliable identification of a broad range of compounds likely to be contained in all types of formulations of explosive devices.

From a practical forensic point of view, the detection technique employed to characterize an explosive material should be capable of a fast, real-time, and highly accurate analysis that does not involve complex sample preparation. These criteria are fulfilled both by the use of ambient ionization sources such as DART (Direct Analysis in Real Time) [2, 3], coupled to mass spectrometry (MS) and by the use of micro-Raman spectroscopy. Recent studies have shown the great potential of Raman spectroscopy (a nondestructive technique, although an excessive incident power may thermally degrade the sample) for identifying structural and compositional information pertaining to explosives samples [4–7]. An advantage of Raman spectroscopy is its ability to directly probe a sample without the need for chemical or mechanical pretreatment of the specimen. Hence, it has been proposed as an initial “primary” screening technique for wipe samples where the presence of explosives is suspected so that follow-up analyses can be subsequently carried out using more sophisticated techniques resulting in minimal sample disruption and consumption of valuable material. The combined usage of DART-MS and Raman spectroscopy can provide separate, high quality and high resolution spectral fingerprints that are orthogonal in nature [8, 9]; each can be employed for identification at trace levels.

Here we report the combined use of ambient mass spectrometry (DART-Orbitrap MS) and micro-Raman spectroscopy for the detection, comprehensive characterization, and differentiation of explosive particles collected on wipe and swab surfaces by a vacuum suction-impact collection device (vacuum impactor) for forensic applications. A series of samples were examined using cotton swabs and wipes that collected various plastic and nonplastic explosives originating from a variety of mines, rockets, mortars, and submunitions.

## Materials and methods

### Sample collection, preservation, and treatment

Swabs and wipes from different origins and fabrications were collected manually from defused explosive devices. The samples are listed in Table 1. The swabs and wipes were placed in a plastic bag that was immediately sealed to protect the samples from potential contamination and stored in the dark at 4 °C.

**Table 1:** Samples collected and analyzed

Sample no.	Device
1	Mine
2	Dispersed explosive
3	Submunition
4	Submunition
5	Submunition
6	Rocket
7	Rocket
8	Rocket
9	Rocket
10	Grenade
11	Mortar 60 mm
12	Mortar 60 mm
13	Mortar 81 mm
14	Mortar 82 mm
15	Bounding mine

**Fig. 1:**
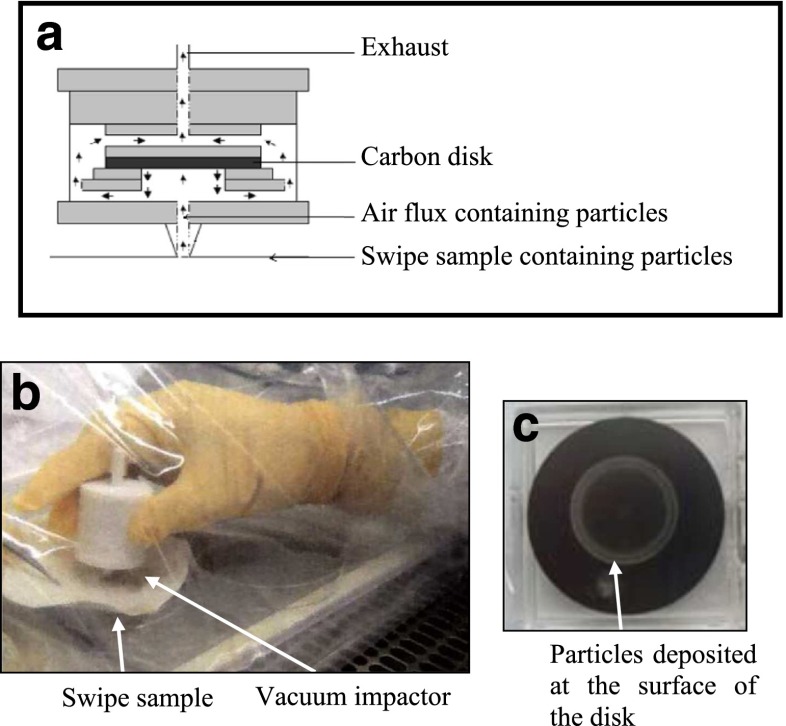
**a** Setup of the vacuum impactor. **b** Sampling using a vacuum impactor on the surface of a swipe sample. **c** After using the vacuum impactor, the carbon disk contains a ring rich in particles collected from the swipe

To collect energetic materials from the wipe samples, we used a vacuum suction-impact collection device that was originally designed to collect uranium particles on an impaction plate made of carbon [10]. Briefly, particles in the wipe sample are driven by vacuum pumping at a flow rate of approximately 2.0 L/min and collected onto the carrier (carbon disk) by impaction. A schematic drawing of the vacuum collector apparatus is displayed in Fig. 1.

### DART ion source conditions

A DART-SVP ion source (Ion Sense, Saugus, MA, USA) interfaced to a LTQ-Orbitrap XL mass spectrometer (Thermo Scientific, San Jose, CA, USA) was used to acquire all mass spectra. Unless otherwise noted, the DART settings were nitrogen/helium gas pressure, 80 psi; gas temperature, 250–300 °C; grid electrode voltage, 200 V; discharge needle voltage, +1.5 kV for positive ion mode or −1.5 kV for negative mode. High-purity nitrogen (99.998 %) was used as the standard gas and the gas was automatically switched to high-purity helium (99.998 %) in “run” mode. A Vapur® (Ion Sense) evacuated flange was located between the DART ion source and the mass spectrometer. A small membrane pump (Vacuubrand, Wertheim, Germany) was used to create a vacuum in the flange. The DART source was oriented such that its outlet was in line with the ceramic tube leading to the flange before the inlet of the mass spectrometer. For some experiments, the DART-SVP source was fitted with a motorized linear rail (transmission mode module). In these experiments, the samples were suspended on a stainless steel mesh and the heated DART ionizing gas passed through the porous sample support enabling efficient ionization of analytes. A constant speed of 2.0 mm/s was used for the motorized linear rail. Chloroform vapors were delivered at a constant flow rate in the helium metastables, between the DART source and the entrance of the mass spectrometer, using the experimental setup shown in Fig. S1 in the Electronic Supplementary Material (ESM). Briefly, a sparger placed in a volumetric flask containing 200 mL of liquid chloroform, and connected to a compressed air gas cylinder, produced chloroform vapors that were delivered to the DART source region using the tip of a pipette.

### Mass spectrometry

High resolution (HR) mass spectral measurements were performed on a LTQ-Orbitrap XL mass spectrometer. The linear ion trap mass spectrometer settings were capillary voltage, 30 V; tube lens voltage, 100 V; capillary temperature, 200 °C. The ion optics settings were as follows: multiple 1 offset voltage, −4.5 V; multiple 2 offset voltage, −8.0 V; lens 1 voltage, −4.2 V; lens 2 voltage, −15.0 V; gate lens voltage, −35.0 V; front lens voltage, −5.25 V. The mass range typically acquired was *m*/*z* 50–1200. The instrument *m*/*z* values were calibrated using the manufacturer’s mixture consisting of caffeine, MRFA (l-methionyl-arginyl-phenylalanyl-alanine acetate monohydrate), and Ultramark 1621. Resonant excitation was carried out using collision-induced dissociation (CID); the LTQ was set to sum 3 microscans, activation time of 30 ms; normalized collision energies (NCE) were between 5 and 30 %, and the precursor ion isolation window was set at 1.0 *m/z* for all investigated compounds. The ion trap collision cell was supplied with ultra-high purity (99.999 %) helium gas. All data analysis was performed using Thermo Xcalibur™ software. Accurate mass measurements, including those of product ions, were performed at high resolution (resolving power of 60,000 FWHM at *m/z* 400).

### Micro-Raman spectroscopy

The MRS (micro-Raman spectroscopy) instrument used in this study (Renishaw PLC ‘InVia’, Wotton-Under-Edge, Gloucestershire, UK) was equipped with two lasers: 514 nm (argon gas) and 785 nm (diode semiconductor) with maximum powers of 50 and 300 mW, respectively. Irradiation time, spectral range, and laser power can be easily adjusted to find the best analytical conditions for each compound. Different objectives can be used for laser focusing (×5, ×20, ×50, and × 100). The × 100 objective is preferably used for optimal focusing and obtaining the thinnest beam, with a diameter close to 1 μm. Calibration of the micro-Raman instrument was carried out using the 520.5 cm^−1^ band of a Si wafer. Data acquisition was carried out with the Renishaw WIRE software. Spectra were not corrected for background.

## Results and discussion

### Collection of explosive particles using a vacuum-impaction device

This work aims to develop a method that uses micro-Raman spectroscopy in combination with a DART mass spectral approach for the analysis of swab and wipe samples collected for the screening of explosives. The investigated samples are listed in Table 1. Our workflow of sample preparation intentionally excluded the time-consuming steps that would normally be required for either lixiviation of the analytes from the wipes and swabs or solid-phase extraction to separate the compounds from the matrix components prior to LC–MS analysis. Rather, we chose to use a vacuum impactor (Fig. 1) to collect micrometric explosive particles from the surface of the swab and wipe samples on an impaction plate made of carbon [10]. The transfer of particles from a wipe or swab sample to the impaction plate lasts only about 10 min. After sample collection, the carbon impaction plate was directly analyzed by micro-Raman spectrometry and then exposed directly to the metastable helium atoms (heated to 350 °C) of the DART ionization source coupled to an LTQ Orbitrap mass spectrometer. This study builds upon a small number of previous investigations that have demonstrated the potential of DART-MS for the analysis of reference explosive compounds spiked in various matrices [2, 3, 11]. Moreover, we recently showed that DART hyphenated to an Orbitrap mass analyzer permitted the direct and rapid characterization of synthetic and natural homopolymers whose molecular weights fall between 200 and 4000 Da [12]. The approach provided useful information concerning polymer compositions such as molecular weight distributions, the nature of terminal functionalities, and oligomeric sequences.

### Raman analysis of explosives on vacuum impactor

Raman spectroscopy provides a unique molecular spectral fingerprint, with each spectrum containing key signature bands that can be used for identification. All Raman spectra were acquired in the range 100–3200 cm^−1^ to enable observation of most Raman bands for common explosives [5–7]. Raman microscopy was applied to focus the laser beam on micrometric targets and collect the Raman scattering from the explosive crystals collected by vacuum impactor directly onto a carbon disk. The NIR laser at 785 nm gave good-quality spectra of explosives, with limited background fluorescence.

**Fig. 2:**
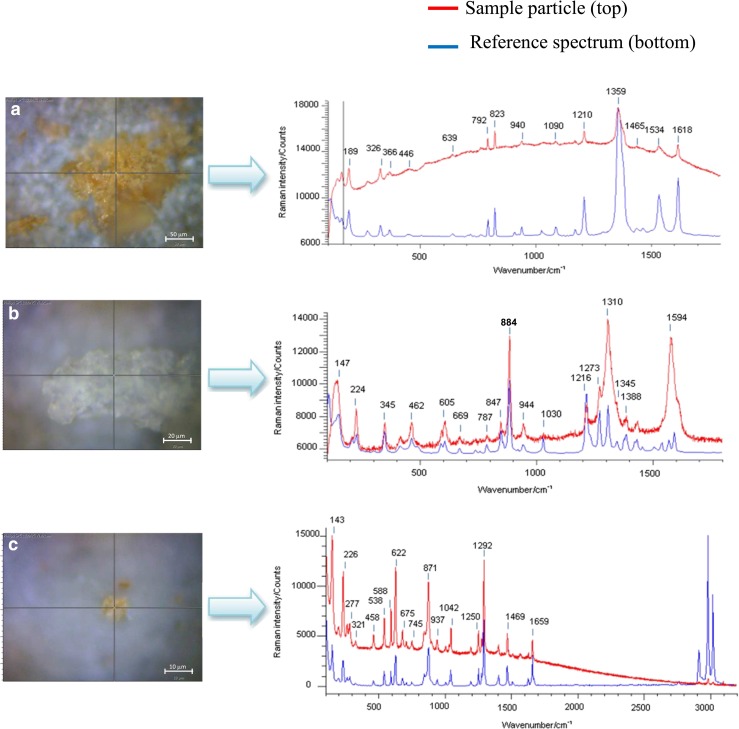
Raman microscopy image of energetic particles collected from the surface of **a** sample 3, **b** sample 4, and **c** sample 10 by vacuum impactor with their respective Raman spectrum in *red*. Raman conditions: 785 nm, 10 s exposure time, 5 accumulations. The reference Raman spectrum from the Renishaw library is overlaid is each case (*blue line*). See ESM Fig. S1 for the Raman spectrum of a blank reference carbon stub

Figure 2a displays the Raman microscopy image of a cluster of energetic crystals collected from the surface of sample 3 (wipe of a submunition). Crystal sizes are typically between 10 and 50 μm. The wavenumbers and relative intensities of peaks observed in the acquired Raman spectrum, represented in red, match well with those of the reference Raman spectrum of trinitrotoluene (TNT) from the Renishaw library (overlaid in blue). The observed peaks can be attributed to the vibrational modes of TNT [4, 5, 7, 13, 14], including characteristic symmetric NO_2_ stretching vibrations at approximately 1360 cm^−1^ and the asymmetric NO_2_ stretching vibrations at 1534 and 1618 cm^−1^. The shoulder at 1380 cm^−1^ may be assigned to the –CH_3_ symmetric bend, and its presence sets TNT apart from most of the explosives belonging to the nitro aromatic group. The other peaks can be assigned to the NO_2_ scissoring modes (790, 820 cm^−1^), to the –CH(ring) bending modes (800–1100 cm^−1^), and to the ring-breathing mode (1210 cm^−1^).

Figure 2b displays in red the Raman microscopy image of a crystal collected from the surface of sample 4 (wipe of a submunition). The experimental Raman spectrum obtained from the particle is dominated by a band at 884 cm^−1^ which can be attributed to the symmetric C–N–C ring breathing mode, as well as the band at 944 cm^−1^ which can be assigned to the ring stretching mode and to the N–O deformation; the band at 1278 cm^−1^ can be attributed to CH_2_ scissoring and the characteristic N–N stretch vibration [15]; the band at 1310 cm^−1^ results from –CH_2_ wagging; the band at 1388 is likely the υNO2 symmetric stretching vibration and βCH_2_ scissoring; finally, the band at 1594 cm^−1^ is attributed to the υNO2 asymmetric stretch in nitro amines [15, 16]. The reference Raman spectrum of RDX, obtained from the Renishaw library, is overlaid in blue (Fig. 2b). It is worth noting that the broad peaks observed in the experimental spectrum at 1310 and 1594 cm^−1^ are due to the contribution of the vibrations of the graphite (carbon) support.

Figure 2c displays the Raman microscopy image of a crystal collected from the surface of sample 10 (wipe of a grenade). The experimental Raman spectrum (red trace) is characterized by Raman lines matching well with literature data [7, 17–19] and can be attributed to the vibrational modes of PETN. Indeed, the band observed at 622 cm^−1^ is due to the rocking vibration mode of –ONO_2_ groups; the band at 871 cm^−1^ results from the O–N stretching mode, the band at 1042 cm^−1^ from the CH_2_ torsion and the bending of the C–C bond, and finally the bands at 1292 and 1469 cm^−1^ result from the symmetric and asymmetric stretching of NO_2_, respectively.

### DART-HRMS analysis in negative mode

This investigation seeks to develop and apply negative ion mode DART-HRMS methods for the direct analysis of swabs and/or wipes holding explosives. To enable direct DART-HRMS analyses of explosive materials aspirated from swabs or wipes, the carbon impaction plate containing the transferred explosive particles was placed between the DART ion source outlet and the entrance of the LTQ-Orbitrap mass spectrometer. The DART parameters were optimized for each of the nitro-based explosives. We found that the optimal gas stream temperature was 350 °C for most explosives. A priori, we anticipated that the explosives bearing sufficiently acidic protons would yield abundant deprotonated molecules [M–H]^−^, whereas those products bearing very weakly acidic protons would be capable of forming anionic adducts, especially if chloride anions were made available, i.e., [M + Cl]^−^ [20]. To promote the formation of chloride adducts, chloroform vapor was added directly to the ionizing region, using a simple experimental setup where helium was gently bubbled into chloroform (ESM Fig. S2).

Table 2 summarizes the “suspected” composition of some of the samples as well as the results corresponding to the screening of the wipe and swab samples. Among the 15 samples analyzed in this work, four explosives were detected, namely TNT, RDX, HMX, and PETN. For the nitramines RDX and HMX, deprotonated molecules were never detected. We attribute this to the very low acidity of the hydrogens (all equivalent on each of these symmetric molecules). The addition of chloroform to the DART reagent gas allowed the facile generation of chloride adducts for HMX and RDX, as well as for the nitrate ester pentrite (PETN). The ionization efficiencies for these species are thus augmented substantially relative to the case where no chloroform is present. However, the presence of chloroform in the source did not result in the production of abundant chloride adducts for TNT. Rather, TNT was detected as deprotonated molecules, (M − H)^−^, which can be rationalized on the basis of both an increased acidity of available hydrogens, and a lower stability of chloride adducts of the neutral molecule.

**Table 2:** Negative ionization screening in swabs from real samples analyzed by DART-LTQ/Orbitrap

Sample no.	Suspected composition	[TNT–H]^−^	*[RDX + ^35^Cl]^−^	[RDX + NO_3_]^−^	*[HMX + ^35^Cl]^−^	[HMX + NO_3_]^−^	[PETN-H]^−^	*[PETN + ^35^Cl]^−^	[PETN-NO_3_]^−^
1	Unknown	–	–	–	–	–	–	–	–
2	Cyclotol ^a^	+	+	+	–	–	–	–	–
3	Tolite ^b^	+	–	–	–	–	–	–	–
4	Unknown	–	+	+	–	–	–	–	–
5	Unknown	+	+	+	+	+	–	–	–
6	RDX + aluminum	–	+	+	–	–	–	–	–
7	Trotyl ^c^	+	–	–	–	–	–	–	–
8	Tolite	+	–	–	–	–	–	–	–
9	Trotyl	+	–	–	–	–	–	–	–
10	Unknown	–	–	–	–	–	+	+	+
11	Unknown	+	–	–	–	–	–	–	–
12	Trotyl	+	–	–	–	–	–	–	–
13	Trotyl	+	–	–	–	–	–	–	–
14	Amatol ^d^	+	–	–	–	–	–	–	–
15	Unknown	–	+	+	+	+	–	–	–

“+”considered ion was detected in the mass spectrum, “–” the ion was absent

^a^ Mixture of RDX and TNT

^b^ TNT-based weapon

^c^ TNT-based weapon

^d^ Military explosive containing TNT and ammonium nitrate

*^37^Cl adduct was observed for all adduct containing ^35^Cl

**Fig. 3:**
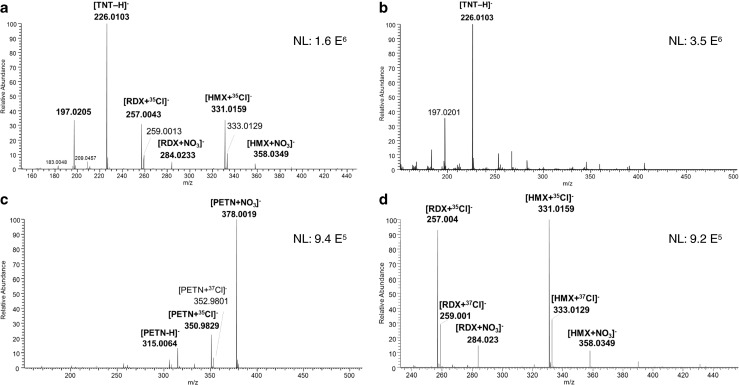
Negative DART-HRMS mass spectra of **a** sample 5 (submunition), **b** sample 9 (rocket), **c** sample 10 (grenade), and **d** sample 15 (bounding mine)

Figure 3 shows representative negative ion mass spectra of samples 5 (submunition, Fig. 3a), 9 (rocket, Fig. 3b), 10 (grenade, Fig. 3c), and 15 (bounding mine, Fig. 3d). In Fig. 3a and b, the [M–H]^−^ peak is readily observed at *m*/*z* 226.0106 for TNT, whereas in Fig. 3a and d, the chloride adduct anions of hexogen [RDX + ^35^Cl]^−^ and octogen [HMX + ^35^Cl]^−^ are observed at *m/z* 257.0043 and *m/z* 331.0159 along with their respective nitrate adduct anions [M + NO_3_]^−^ at *m/z* 284.0233 and *m/z* 358.0350. All peak identifications are consistent with reference mass spectra obtained from pure standards. Deprotonated molecules were not detected for HMX, RDX, or PETN. Neither the nitramines nor the nitrate ester can readily transfer a proton to the strong reagent O_2_^·−^ present in the DART plasma which has been postulated to become protonated to form · O_2_H, a species having a low gas-phase acidity [21]. However, adding chloroform as dopant to the heated helium gas produced abundant Cl^−^ ions that readily formed anionic adducts with HMX, RDX, and PETN. The ability to observe highly abundant chloride adducts has been linked to an exceptional stability of these species in the gas phase [22].

Further DART-MS screening revealed that the three investigated submunition weapons (samples 3–5) did not contain the same explosives. For example, only TNT was detected in sample 3, whereas sample 4 revealed only RDX (Table 2). Other samples, however, contained mixtures of energetic nitro-based charges. For example, analysis of sample 5 by DART-MS revealed a mixture of HMX, RDX, and TNT, as deduced from the detection of [TNT–H]^−^ at *m/z* 226 and the RDX adducts [RDX + ^35^Cl]^−^ and [RDX + NO_3_]^−^ anions at *m*/*z* 257 and 284, respectively. In the case of the rocket weapons, only sample 6 contained RDX, whereas TNT was detected in samples 7–9. No differences were found in mortar weapons (samples 11–14) where only TNT was detected, and its structure is confirmed by the presence of the fragment ion *m/z* 196 formed under CID by loss of CH_2_O [23]. On the other hand, sample 2 (dispersed explosive) contained a mixture of RDX and TNT, which can be attributed to the presence of “Composition B” [24], a mixture that is commonly used as explosive filling in mines. By comparison, the analysis of the bounding mine sample (no. 15) also revealed a mixture containing RDX, but this time the other main component was HMX. The analysis of the grenade swab (sample 10) revealed uniquely pentaerythritol tetranitrate (PETN), detected in three different forms: *m/z* 315.0064 [PETN–H]^−^, *m/z* 351.9830 [PETN + ^35^Cl]^−^, and *m/z* 378.0019 [PETN + NO_3_]^−^. All samples containing TNT displayed an abundant ion at *m/z* 197, whose signal intensity varied among the samples. The interpretation of this unassigned ion is discussed in the ESM.

All weapon samples were swiped several times, and each swipe was analyzed at least three times (three replicates) in different areas of the swipes. Although the relative intensity of the analytes detected in each replicate varied within a range of ±30 %, each replicate analysis revealed the same composition.

### DART-HRMS analysis in positive mode

The analysis of explosives in the positive ion mode provides additional and valuable information for the characterization of explosives that complements that obtained in the negative ion mode. The latter is often used to ionize nitroaromatic-based explosives; however, analysis in positive ion mode can reveal the binder polymers used in manufacturing. These polymers are added to “phlegmatize” the main charge, i.e., they stabilize or desensitize the explosive. Other informative compounds can also be detected in positive ion mode, such as nonexplosive organic compounds, degradation products, plasticizers, and tagging agents.

Various polymers are commonly used as explosive fillers as well as plasticizers and binders in energetic materials. The use of these compounds in explosive formulations is mainly for desensitization purposes; however, with the development of more insensitive explosive fillers, the function of the polymer binder is shifting from that of a desensitizer to that of providing structural integrity [25]. Intact synthetic polymers are widely analyzed by matrix-assisted laser desorption/ionization mass spectrometry (MALDI-MS) [26, 27], but this method is limited by the sensitivity when trace analysis is required. ESI-MS is another useful technique for the analysis of polymers, and it has the advantage that it can be easily interfaced with liquid separation methods (e.g., HPLC, CE). Some disadvantages of ESI are the tendency for signal suppression of less surface-active species when mixtures or co-eluting species are present, and the strict solvent requirements [28]. Alternatively, direct analysis of polymers using ambient ionization techniques has become useful [29], providing analysis methods which are fast and without special sample preparation requirements. In this way, DESI-MS was used for polymer analysis and showed good performance for the analysis of industrial synthetic polymers [30–32]; however, like ESI, DESI provides multiply charged species whose presence may complicate the mass spectral profile. Another direct sampling technique, DART-MS, provides only singly charged species, and when coupled to high-resolution instruments, represents a powerful tool for the analysis of polymers [12]. Thus, DART-MS has been demonstrated to be fast and nondestructive [33], and has utility for the analysis of synthetic and natural polymers [12].

**Fig. 4:**
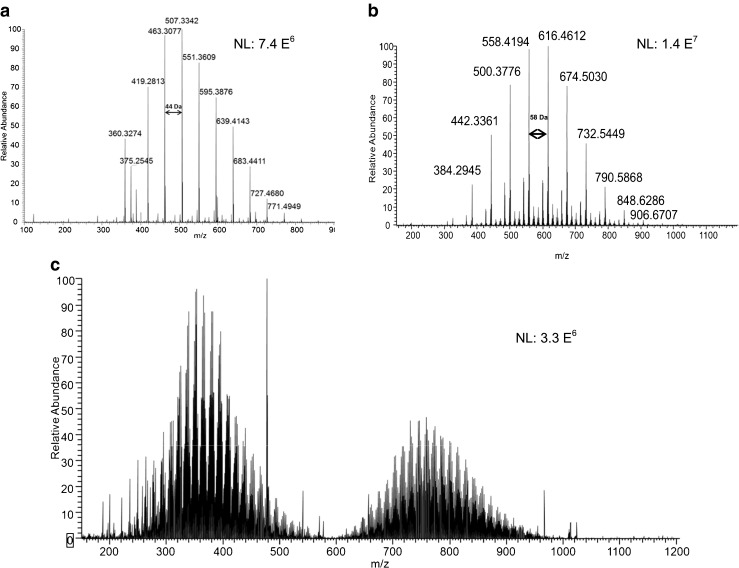
Mass spectra of the screening in positive ion mode by using DART-MS: **a** PEG polymer in rocket sample 8, **b** PPG polymer in grenade sample 10, **c** unknown (wax-like) compounds found in bounding mine sample 15

In the current study, the analysis of explosive samples collected on an impaction plate using DART-MS produced only singly charged species. For most samples, the analysis of swabs showed the presence of polymers. Specifically, samples 3–9 and 11–14 all showed a low molecular weight polymer distribution, which was separated by a 44-Da repeat unit which we assign as ethylene oxide (−OCH_2_CH_2_–) characteristic of polyethylene glycol (PEG) polymer, as exemplified in Fig. 4a. This mass spectrum showed singly protonated [M + H]^+^ molecules and singly charged ammonium adducts [M + NH_4_]^+^. Ammonium adducts likely arise from ammonium salts present in the samples, or present in residual amounts on the surface of the ionization source, because no ammonium compounds were added to the heated helium stream. Figure 4b displays the mass spectrum of sample 10 (grenade) showing singly charged species [M + NH_4_]^+^ and [M + H]^+^. The spectrum shows a series of ions separated by 58 *m/z* units, which we assign to the propylene oxide (PO) repeat unit (−OCH_2_CH_2_CH_2_–) corresponding to polypropylene (or isopropylene) glycols (PPG). Unlike the other samples analyzed, sample 15 shows a very complex mass spectrum displayed in Fig. 4c. Although DART-MS produces only singly charged species, the polymeric sample contains variable repeat units, and the number of possible combinations leads to a complex interpretation of the mass spectrum. Furthermore, the small quantity of sample did not allow us to perform additional experiments (e.g., MS/MS, FTIR, etc.), which can provide complementary information for structural elucidation and identification.

**Fig. 5:**
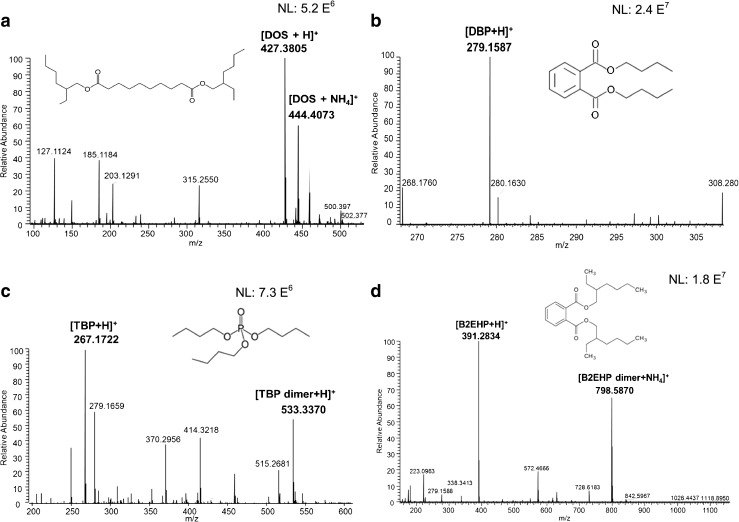
Mass spectra of samples acquired in positive mode using DART-LTQ/Orbitrap: **a** plasticizers from sample 1 (mine); **b**–**d** plasticizers from sample 14 (mortar)

On the other hand, sample 1 (mine) did not show any polymer constituents. However, a plasticizer was detected at *m/z* 427 corresponding to protonated [M + H]^+^ diisooctyl sebacate (DOS) and its ammonium adduct [M + NH_4_]^+^ was observed at *m/z* 444 (Fig. 5a). Other plasticizers were observed, such as tributyl phosphate (at *m/z* 267, corresponding to the protonated molecule and *m/z* 533, resulting from the dimerization of TBP, Fig. 5b); dibutyl phthalate ([M + H]^+^ observed at *m*/*z* 279, Fig. 5c); and bis(2-ethylhexyl)phthalate ([M + H]^+^ at 391, Fig. 5d). Plasticizers are critical components in explosive formulations. These compounds are used in explosive formulations to alter the mechanical characteristics of the polymer binders by increasing the flexibility of the polymer chain. In addition to improving the mechanical properties, plasticizers also tend to reduce the mix viscosity to improve processing, modify the oxygen balance, and modify the burn rate characteristics of the energetic material composite [26].

## Conclusion

We have demonstrated a novel approach to collect and characterize explosive particles from real military explosives sampled by wiping surfaces with cotton swabs and wipes. This approach relies on the use of a vacuum impactor for the transfer of micrometer-size explosive particles from the wipes to an impaction plate made of carbon. The particles deposited on the carbon plate are then characterized using Raman microscopy followed by DART mass spectrometry, thereby providing spectral fingerprints that are orthogonal in nature. Indeed, Raman microscopy provides an efficient way to screen for the detection and identification of the explosive charge on the impaction plate. Further, we showed that the resolving power (60,000 FWHM at *m/z* 400) and mass accuracy (<2 ppm) of DART-MS used in both positive and negative ionization modes allowed the global characterization of plastic and nonplastic explosive formulations at trace levels. In fact, the natures of the explosive charges (TNT, RDX, HMX, and PETN) were identified as either deprotonated species or as nitrate or chloride adduct anions in negative mode. Switching to a positive mode of ionization revealed the presence of polymer binders, plasticizers, and other additives identified either as protonated or ammonium adduct species. In our laboratory, the DART/Orbitrap method produced LODs in the picogram range for TNT, PETN, and Tetryl and nanogram range for HMX, PETN, and Tetryl directly analyzed on the swabs and swipes (data not shown), confirming that DART/MS is a method that can be used for detection and confirmation of trace amounts of explosives. Although no detection limit was calculated in this study for the micro-Raman method, recent studies reported using Klarite® substrates to collect surface-enhanced Raman scattering (SERS) spectra of explosives (i.e., TNT) down to 200 pg, quantities suitable for detection of explosives at the trace level [7].

These results clearly demonstrate the capability of Raman microscopy combined with DART-MS as a tool for the fast screening, comprehensive characterization, and differentiation of particulate explosive samples for forensic sciences and homeland security applications. Future work will focus on analyzing the same types of explosive residues on samples collected post blast.

## Electronic supplementary material

Below is the link to the electronic supplementary material.

ESM 1 (PDF 591 kb)
