# Supplementary material for: Combined use of direct analysis in real-time/Orbitrap mass spectrometry and micro-Raman spectroscopy for the comprehensive characterization of real explosive samples
Source: Anal Bioanal Chem. 2016 Jun 18;408:5677–87. doi: 10.1007/s00216-016-9691-9 (PMC4958401; doi:10.1007/s00216-016-9691-9)
Supplement: Supplementary file 1 — (PDF 591 kb) [file 216_2016_9691_MOESM1_ESM.pdf]

Analytical and Bioanalytical Chemistry

Electronic Supplementary Material

Combined use of direct analysis in real-time / orbitrap mass spectrometry and micro-Raman spectroscopy for the comprehensive characterization of real explosive samples

Maxime C. Bridoux, Adrián Schwarzenberg, Sébastien Schramm, Richard B. Cole

Interpretation of the unassigned ion at m/z 197, present in all the samples containing TNT.

An ion at *m/z* 197 has previously been observed by other groups that used MS to characterize TNT [3,22]. In order to investigate the structure of this unassigned ion, accurate mass measurements were performed. The elemental composition of the ion appearing at *m/z* 197.0205 is $[C_7H_5O_5N_2]^-$ (Δ_{ppm} 0.6, RDB 6.5). Moreover, collision induced decomposition (CID) was employed to obtain structural information concerning this m/z 197 precursor ion. CID of *m/z* 197.0205 produced the fragment ions at *m/z* 180.0178 $[C_7H_4O_4N_2]^-$, *m/z* 167.0226 $[C_7H_5O_4N]^-$ and *m/z* 137.0245 $[C_7H_5O_3]^-$ as displayed in **Figure S3**. The fragment ion at *m/z* 180.0178 is assigned to the release of $OH^•$ which we are attributing to the "*ortho* effect", and it allows us to postulate the structures of m/z 197.0205 shown in Scheme S1 (Supplemental information). Further, fragment ions *m/z* 167.0226 and *m/z* 137.0245 were obtained by consecutive losses of $NO^•$ and 2 $NO^•$ respectively, confirming the presence of two nitro groups. Furthermore, high resolution m/z measurements revealed that the fragment ion at *m/z* 167 was actually comprised of two ions as shown in **Figure S3**, where the first, and more abundant ion (*m/z* 167.0226) corresponds to a loss of $NO^•$, whereas the second ion in lower abundance (*m/z* 167.0101) results from the loss of CH_2O. The two pathways leading to these two fragment ions are competitive, and the products are formed in a 3:1 ratio. This behavior was previously observed and explained by Schwarzenberg et al [26], when the TNT degradation product 2-amino-4,6-dinitrotoluene (2A-4,6-DNT) was analyzed by ESI-HRMS (electrospray high resolution mass spectrometry). It was concluded that an electron-withdrawing group in the *para* position yields a competitive fragmentation between $NO^•$ and CH_2O in a 3/1 ratio, whereas an electron-donor group in *para* favored the NO_2/ONO isomerization prior to dissociation giving only the loss of $NO^•$. Our assignment of the -OH group in the *ortho* position and the $-NO_2$ group in *para* position of the toluene ring, is thus further rationalized (Scheme S1). To our knowledge, this ion does not correspond to a known explosive or additive used in weapons formulations.

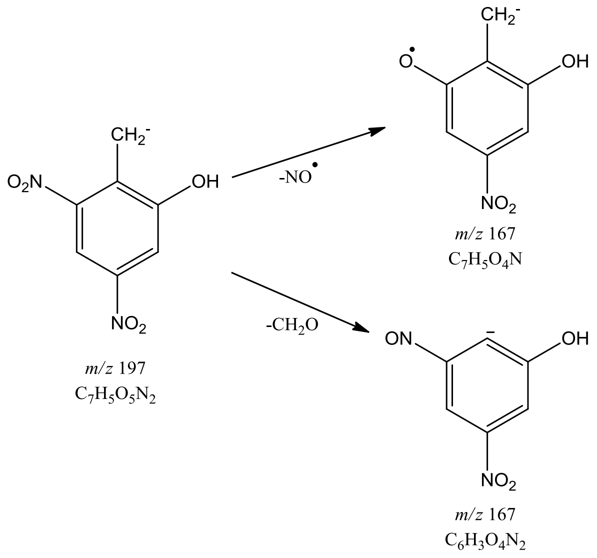

Scheme S1 Proposed structure and fragmentation pathways for the unknown ion m/z 197

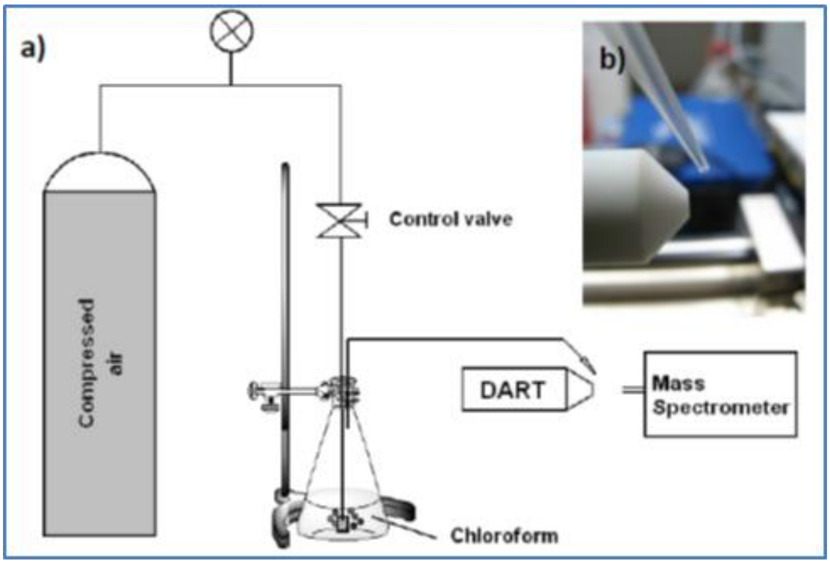

Fig. S1 Scheme of experimental set-up used to introduce chloroform vapors into the stream of helium metastables emanating from the DART source

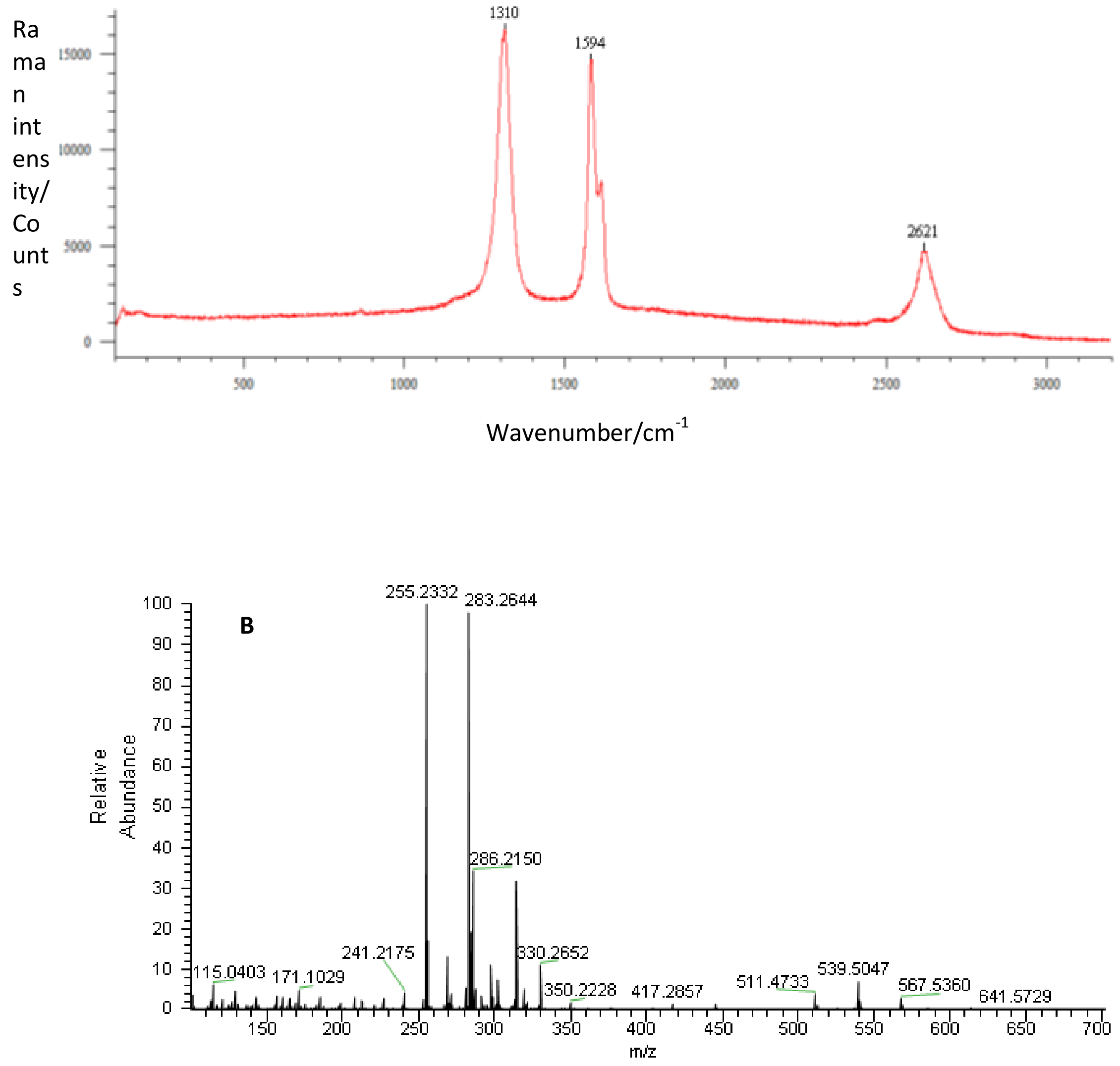

Fig. S2 A) Raman spectrum of a reference carbon stub. Raman conditions: 785 nm, 10s exposure time, 5 accumulations; B) DART-HRMS of a reference carbon stub

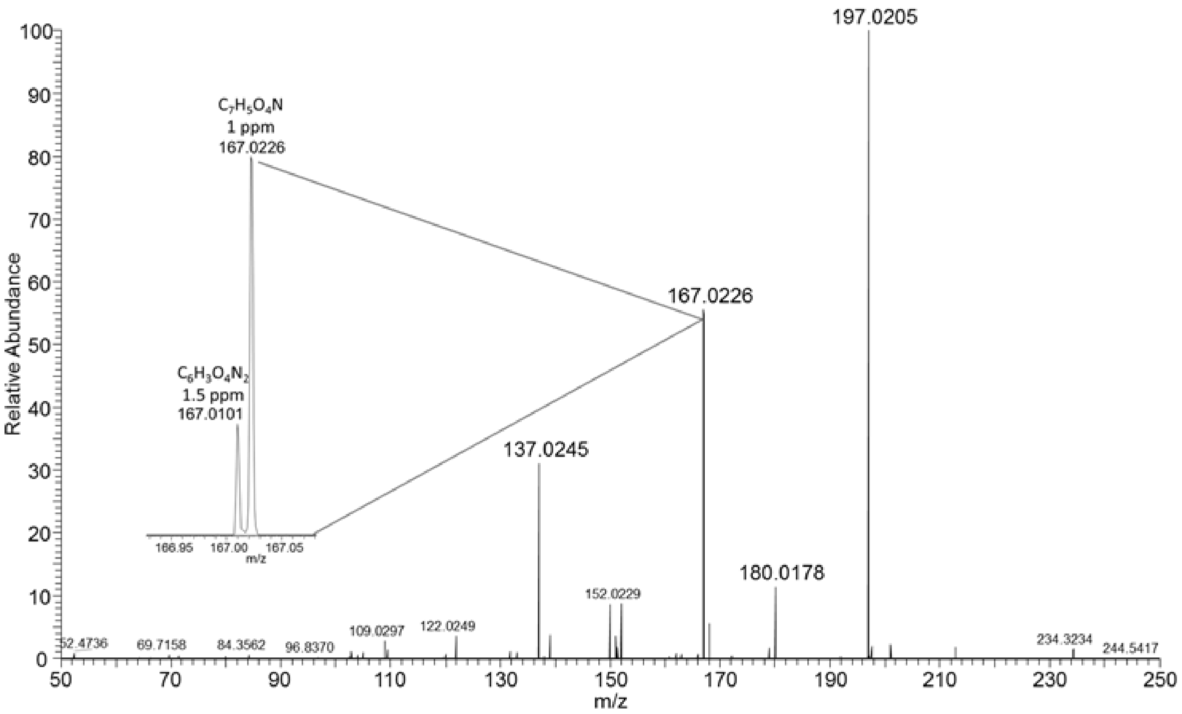

Fig. S3 CID spectrum of the unknown *m/z* 197 at 20% NCE
